# CD27 signaling inhibits tumor growth and metastasis via CD8 + T cell-independent mechanisms in the B16-F10 melanoma model

**DOI:** 10.1007/s00262-024-03780-9

**Published:** 2024-08-06

**Authors:** Eswara Rao Puppala, Long Wu, Xiaoxuan Fan, Xuefang Cao

**Affiliations:** 1 Marlene and Stewart Greenebaum Comprehensive Cancer Center, University of Maryland Baltimore School of Medicine, Baltimore, MD 21201 USA; 2 Department of Microbiology and Immunology, University of Maryland Baltimore School of Medicine, Baltimore, MD 21201 USA

**Keywords:** B16-F10 melanoma, CD27, CD8 + T cells, Tumor growth, Tumor metastasis

## Abstract

CD27 belongs to the tumor necrosis factor receptor superfamily and acts as a co-stimulatory molecule, modulating T and B cell responses. CD27 stimulation enhances T cell survival and effector functions, thus providing opportunities to develop therapeutic strategies. The current study aims to investigate the role of endogenous CD27 signaling in tumor growth and metastasis. CD8 + T cell-specific CD27 knockout (CD8Cre-CD27fl) mice were developed, while global CD27 knockout (KO) mice were also used in our studies. Flow cytometry analyses confirmed that CD27 was deleted specifically from CD8 + T cells without affecting CD4 + T cells, B cells, and HSPCs in the CD8Cre-CD27fl mice, while CD27 was deleted from all cell types in global CD27 KO mice. Tumor growth and metastasis studies were performed by injecting B16-F10 melanoma cells subcutaneously (right flank) or intravenously into the mice. We have found that global CD27 KO mice succumbed to significantly accelerated tumor growth compared to WT controls. In addition, global CD27 KO mice showed a significantly higher burden of metastatic tumor nests in the lungs compared to WT controls. However, there was no significant difference in tumor growth curves, survival, metastatic tumor nest counts between the CD8Cre-CD27fl mice and WT controls. These results suggest that endogenous CD27 signaling inhibits tumor growth and metastasis via CD8 + T cell-independent mechanisms in this commonly used melanoma model, presumably through stimulating antitumor activities of other types of immune cells.

## Introduction

The immune system has the capacity to protect against tumor development and progression, and immune based therapy has become effective for more and more cancer patients. The CD27-CD70 pathway belongs to the TNF receptor superfamily that plays important roles in both innate and adaptive immune cells. The interaction between CD27 and its ligand, CD70, is involved in lymphoid differentiation, proliferation, and activation [1]. CD27 was first identified as an important co-stimulatory receptor present on a large subset of peripheral T lymphocytes and most medullary thymocytes [2]. Stimulation of CD27 by its antibodies along with TCR/CD3 stimulation led to enhanced T cell proliferation [2]. Subsequent studies performed with CD27-deficient and CD70-transgenic mice have defined a non-redundant role of this receptor-ligand pair in shaping adaptive T cell responses [3]. Furthermore, other studies have expanded the role of CD27-CD70 interaction in regulating cellular activity in subsets of T, B, and NK cells [3]. Due to the immune stimulatory potential, several CD27-targeting strategies, including agonistic mAbs and engineering intracellular domains in CAR-T cells, have been tested in preclinical models and clinical trials targeting solid or hematological malignancies [4, 5].

However, a number of studies have described diverse roles for this pathway in different subsets of immune cells, which could lead to opposing effects on tumor immunity. For example, it was reported that CD27-CD70 interaction increased the frequency of regulatory T (Treg) cells, reduced tumor-specific T cell responses, and promoted tumor growth [6]. CD27 signaling was shown to reduce apoptosis of Treg cells in vivo and enhance IL-2 expression, a key survival factor for Treg cells. Consequently, the frequency of Treg cells and growth of solid tumors were reduced in CD27-deficient mice [6]. Another study also showed that Treg-derived CD27 limited antitumor immunity, and ablation of Treg-expressed CD27 synergized with PD-1 blockade to improve cytotoxic T lymphocyte-mediated tumor control [7]. In contrast, the CD27-CD70 axis was shown to be critical for mediating interaction between conventional type 1 dendritic cells (cDC1s) and adoptively transferred tumor antigen-specific T cells, promoting the expansion and antitumor efficacy of adoptively transferred CD8 + T cells [8], yet it appeared to be dispensable for the antitumor efficacy of neoantigen vaccine and generation of neoantigen-specific CX3CR1 + CD8 + T cells [9]. In the setting allogeneic hematopoietic cell transplantation (allo-HCT), our previous studies showed that CD27-CD70 signaling in the host as well as donor T cells suppressed inflammatory T cell responses that involved both CD4 + and CD8 + T cells [10, 11].

In this context, we aimed to further delineate the role of CD27 signaling in CD8 + T cells in tumor immunity. We have developed CD8 + T cell-specific CD27 knockout (CD8Cre-CD27fl) mice by breeding CD8aCre mice with CD27 floxed mice. We used the CD8Cre-CD27fl mice along with the global CD27 KO mice to study how selective CD27 deletion from CD8 + T cells and global CD27 KO affect tumor growth and metastasis in the B16-F10 melanoma model. Our results demonstrate that endogenous CD27 signaling inhibits tumor growth and metastasis via CD8 + T cell-independent mechanisms, presumably through stimulating antitumor activities of other types of immune cells.

## Materials and methods

### Cell line and mice

B16-F10 cells were procured from ATCC (Manassas, VA, USA). Cells were maintained in Dulbecco’s modified Eagle’s medium (DMEM) high glucose media supplemented with 10% fetal bovine serum (FBS) at 37 °C in a 5% CO2 incubator [12]. The global CD27-/- mice were provided by Stephen Schoenberger (La Jolla Institute for Allergy and Immunology) and originally generated by Jannie Borst (Netherlands Cancer Institute) [13]. CD8a^cre^ mice were procured from Jackson Laboratories and bred with CD27 floxed mice to develop the homozygous CD8Cre-CD27^fl/fl^ mice. All the mice were housed in specific pathogen-free ventilated cages throughout the study period. A light–dark cycle of 12 h. and relative humidity of 40–70% were maintained during the study period. Animals have free access to fresh water and a standard pellet diet throughout the study. This study’s experimental procedures complied with the animal care and use guidelines from the Office of Animal Welfare Assurance at the University of Maryland School of Medicine Veterinary Resources and protocols were approved by the Institutional Animal Care and Use Committee.

### Tumor growth experiments

Tumors were developed by injecting 1 × 10^6^ B16-F10 cells subcutaneously into the right flank of the mice. Tumor size was measured using Vernier calipers at least twice a week throughout the experiment [14]. Mice were checked for vitality at least three times each week throughout the study period [15, 16]. Mice were euthanized upon reaching 20 mm tumor size in any dimension. The tumor volume was calculated using the formula below (*V* = tumor volume, *X* = length of the tumor, *Y* = width of the tumor).

$$ V = \, \left( {\left( { \, X^{2} } \right)xY} \right)/2 $$

### Tumor metastasis experiments

To investigate tumor metastasis, 3 × 10^5^ B16-F10 cells were intravenously injected into the mice. At the endpoint, mice were euthanized, and lungs were harvested and fixed in a 4% phosphate-buffered formalin (PFA) solution. The tumor metastatic nodules in the lungs were counted using a Zeiss electron microscope [12].

### Flow cytometry

Cell surface staining was performed following our standard laboratory protocol [12]. Briefly, cell surface markers and LIVE/DEAD fixable aqua were stained together in FACS buffer (PBS + 2% FBS). After 15 min of incubation, cells were washed with wash buffer and fixed overnight at 4 °C using an Intracellular fixation buffer. After fixation, samples were transferred into FACS buffer and analyzed using the Cytek aurora spectral flow cytometer (Cytek Biosciences) in the Center for Innovative Biomedical Resources at the University of Maryland School of Medicine. FlowJo software was used to analyze unmixed samples.

### Statistical analysis

Statistical analysis was performed using GraphPad Prism 8.0 software (GraphPad Software Inc., CA, and USA). Tumor volume values were expressed in Mean ± SEM. Tumor growth curves were analyzed using the two-way ANOVA. Lung tumor nest counts were analyzed using the unpaired T test. Survival curves were analyzed using the Log-rank (Mantel-Cox) test. *P* < 0.05 is considered as statistically significant [17].

## Results

### Generating CD8 + T cell-specific CD27 knockout (CD8Cre-CD27fl) mice

**Fig. 1:**
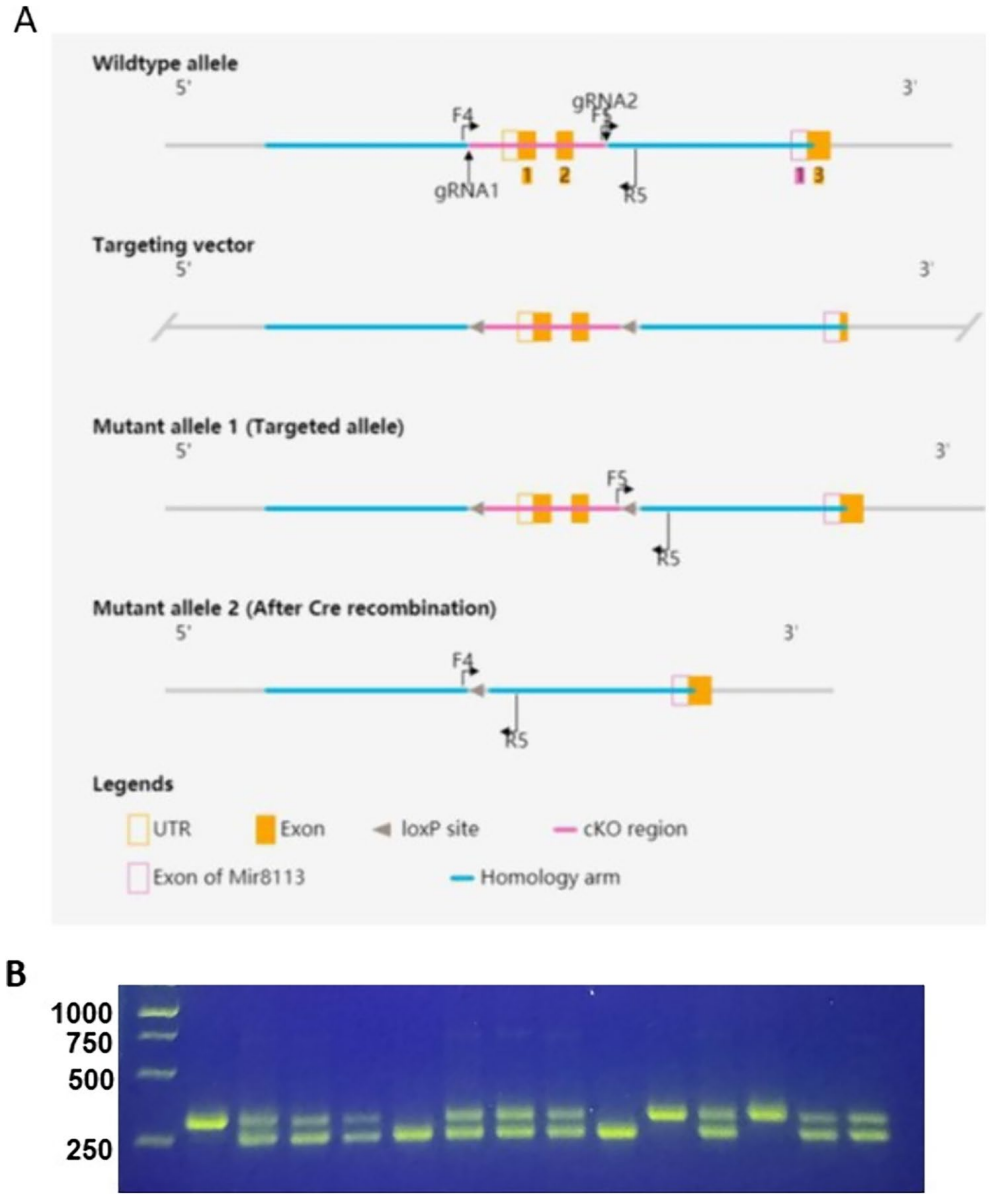
Generating and genotyping CD27 floxed mice. **A** Targeting and genotyping strategy for generating CD27 floxed allele. **B** PCR genotyping results of progenies produced from breeding of heterozygous CD27 floxed mice

We used CRISPR/Cas9-mediated genomic engineering to create a CD27 conditional knockout mouse model in the C57BL/6 J strain (Fig. 1A). Mouse CD27 gene (NCBI Reference Sequence: NM_001033126; Ensembl: ENSMUSG00000030336) is located on chromosome 6. Exons 1–2 were selected as conditional knockout region (cKO region). Deletion of this region results in the loss of function of the mouse CD27 gene. Briefly, Cas9, gRNA and targeting vector were co-injected into fertilized eggs to generate CD27 floxed allele. The resultant progenies were genotyped through polymerase chain reaction (PCR) followed by sequencing analysis, with Forward primer: 5’-TCCATGGATAGGACTCAGACAAC-3’ and Reverse primer: 5’-CTACCATGTGGCCCAGAGAGTAAA-3’. The PCR band sizes are 257 bp for wildtype allele and 314 bp for floxed allele (Fig. 1B).

**Fig. 2:**
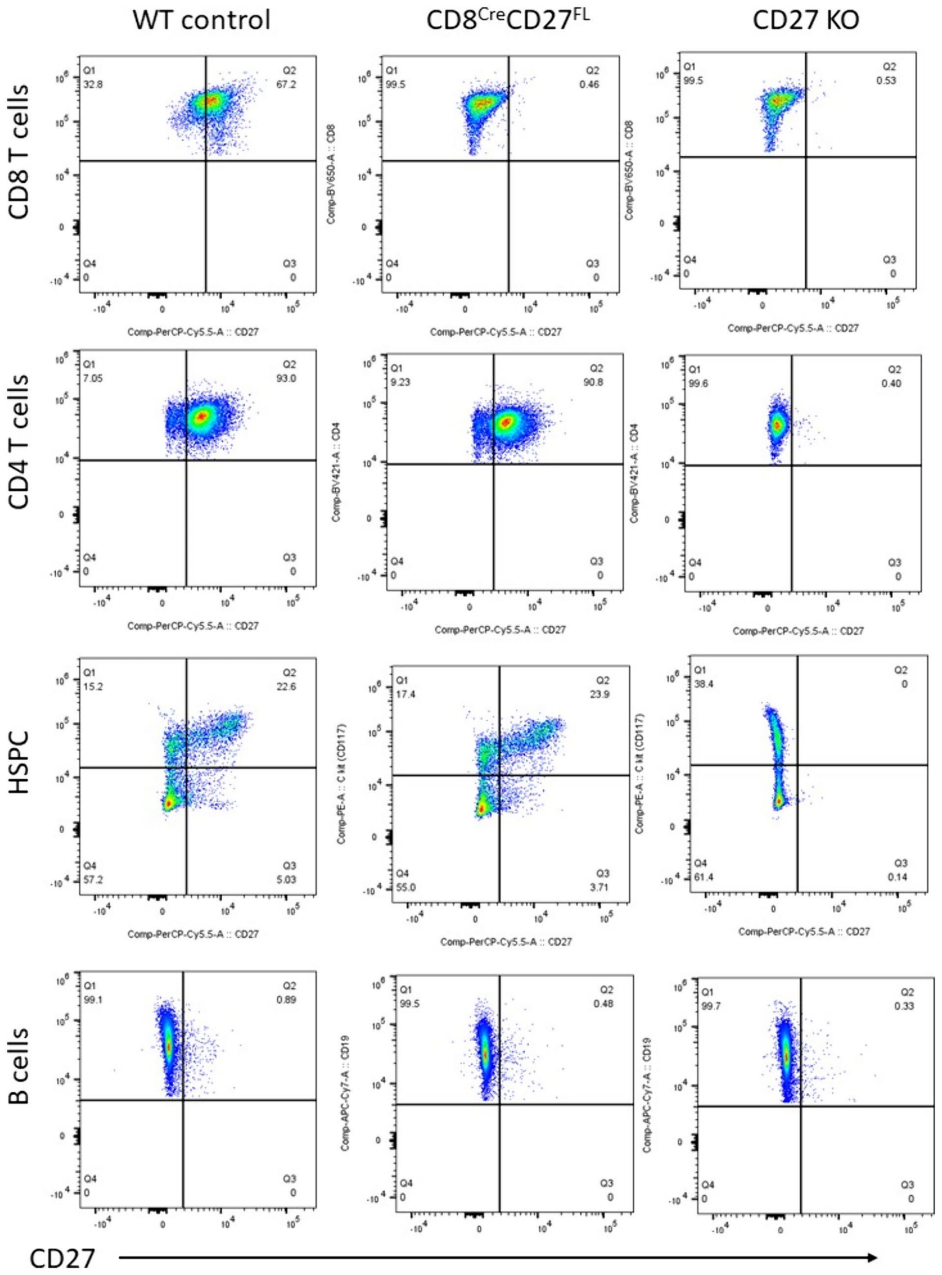
CD8 + T cell-specific CD27 KO (CD8Cre-CD27fl) does not affect CD27 expression in other cell types, while CD27 is deleted from all cell types in global CD27 KO mice. Representative flow cytometry plots show the expression CD27 protein on live CD45 + TCRb + CD8 + and CD4 + T cells, CD45 + CD19 + B cells in the spleen and CD45 + Lineage-CD117 + HSPCs in the bone marrow of the indicated genotypes. Spleen and bone marrow cells were harvested and analyzed via flow cytometry (N = 3 in each genotype for 2 independent experiments)

To delineate the role of CD27 signaling in CD8 + T cells in tumor immunity, we have developed CD8 + T cell-specific CD27 knockout (CD8Cre-CD27fl) mice by breeding CD8aCre mice with CD27 floxed mice. Homozygous CD8Cre-CD27fl mice were established and confirmed by PCR (Fig. 1B). Flow cytometry analyses confirmed that CD27 protein was specifically deleted from CD8 + T cells, but remained unchanged in CD4 + T cells, B cells, and hematopoietic stem/progenitor cells (HSPCs) in the homozygous CD8aCre-CD27fl mice (Fig. 2). We used global CD27 KO mice as controls for flow cytometry analyses, in which CD27 was deleted from all cell types examined. In addition, we have examined CD8 + and CD4 + T cell frequency in thymus and spleen and found no significant changes in either CD8Cre-CD27fl mice or global CD27 KO mice under specific pathogen free condition.

### CD27 inhibits tumor growth in the B16-F10 melanoma model independent of CD8 + T cells

**Fig. 3:**
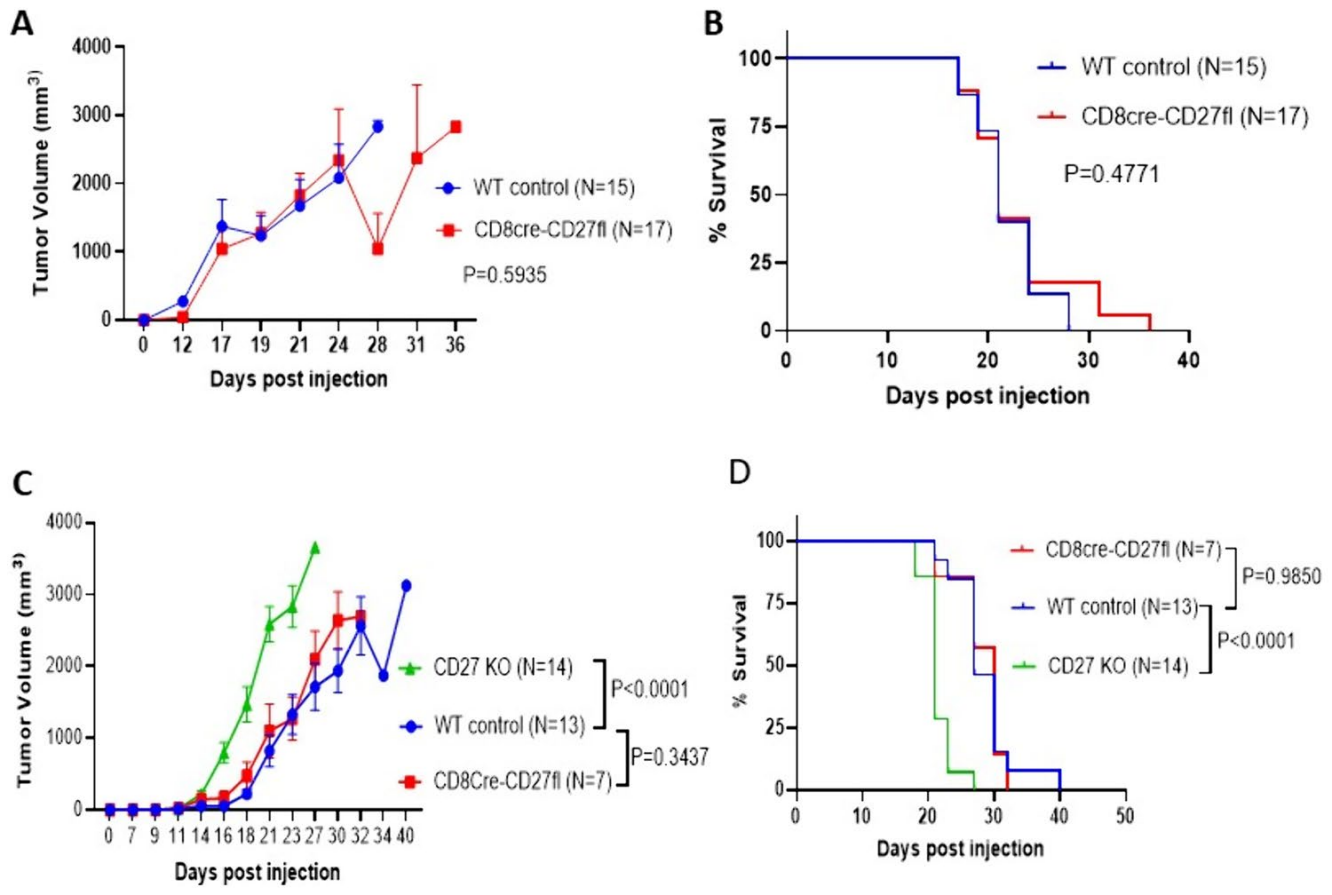
Global CD27 KO results in accelerated tumor growth and lethality in B16-F10 melanoma model, while CD8 + T cell-specific CD27 KO (CD8Cre-CD27fl) shows no effect. 1 × 10^6^ B16-F10 melanoma cells were injected subcutaneously to develop tumors. Tumor size was measured at least twice weekly throughout the experimental period. **A, C** Tumor growth curves from two independent experiments are presented as mean ± SEM. Two-way ANOVA was performed to determine statistical significance. **B, D** Tumor bearing mice were sacrificed when tumor size reached 20 mm in any diameter. Survival curves from two independent experiments were analyzed using the Log-rank (Mantel-Cox) test

After confirming specific deletion of CD27 from CD8 + cells, we sought to investigate the B16-F10 melanoma tumor growth in both global CD27 KO and CD8Cre-CD27fl mice. Briefly, 1 × 10^6^ B16-F10 cells were subcutaneously injected on the flank. After tumors grew to visible sizes, tumor volumes were recorded at least twice a week. A mortality check was done once daily throughout the experiment period. We observed significantly accelerated tumor growth in the CD27 KO group compared to WT controls or the CD8Cre-CD27fl group (Fig. 3C). In contrast, there was no significant difference in the tumor growth curves between the CD8Cre-CD27fl group and WT controls (Fig. 3A–C). Furthermore, the survival curves revealed that CD27 KO mice succumbed to tumor growth in a significantly faster pace compared to WT controls or the CD8cre-CD27fl group (Fig. 3D), while there was no significant difference in the survival curves between the CD8Cre-CD27fl group and WT controls (Fig. 3B–D). These results demonstrate that global CD27 KO results in significantly increased tumor growth, while CD8 + T cell-specific CD27 KO does not affect tumor growth. Therefore, these experiments with the B16-F10 melanoma model indicate that endogenous CD27 signaling in the host inhibits tumor growth via CD8 + T cell-independent mechanisms.

### CD27 suppresses tumor metastasis via CD8 + T cell-independent mechanisms

**Fig. 4:**
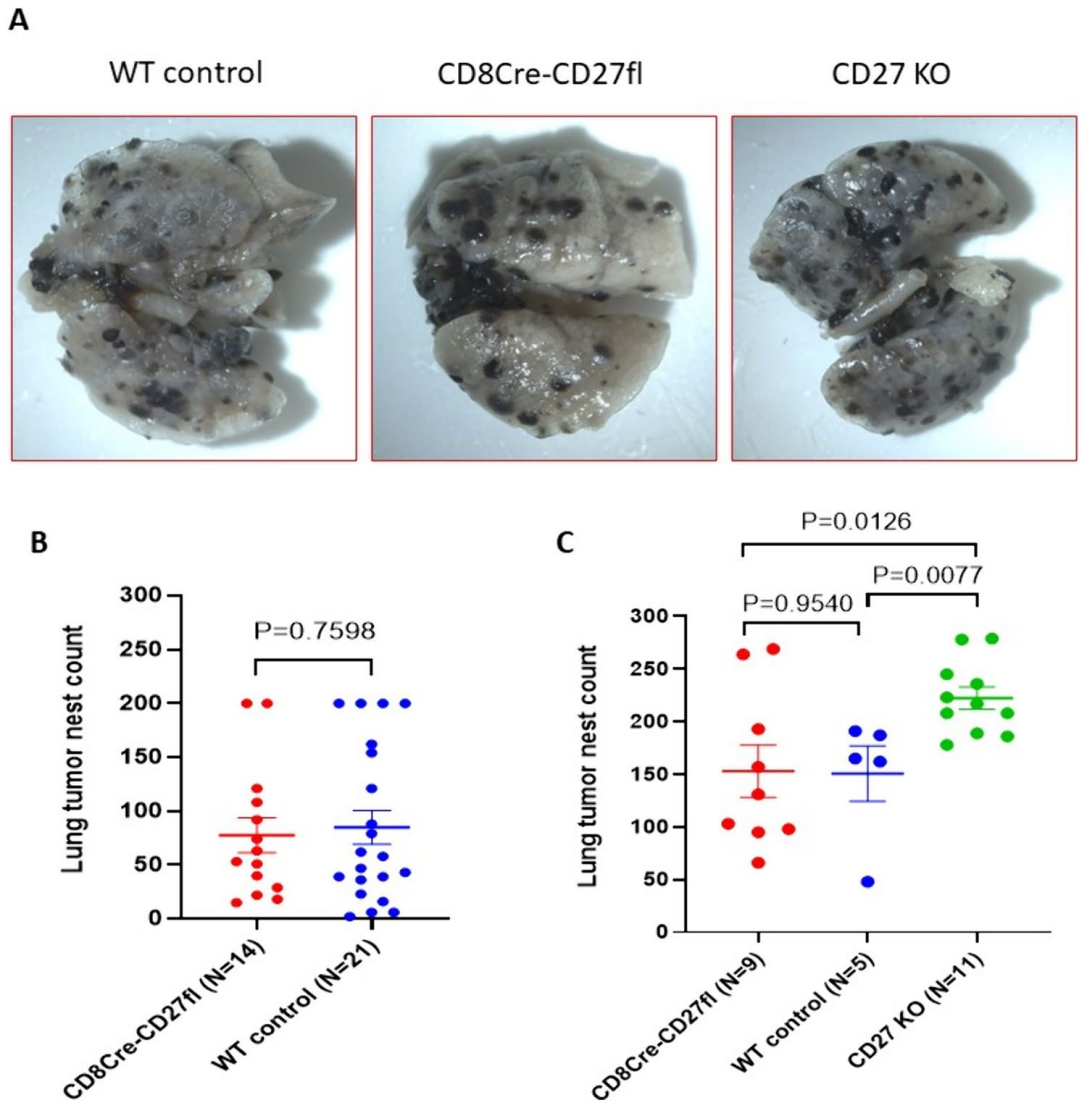
Global CD27 KO results in increased tumor metastasis, while CD8 + T cell-specific CD27 KO (CD8Cre-CD27fl) shows no effect. Lung metastasis was developed by intravenous injection of 3 × 10^5^ B16-F10 cells. **A** Representative lung metastasis images from mice of the indicated genotypes. **B, C** Lung metastasis burdens (tumor nest counts) from 2 independent experiments were presented as mean ± SEM. Lung tumor nest counts were analyzed using the unpaired T test to determine statistical significance

Tumor metastasis burden in the mice was examined by intravenous injection of B16-F10 cells. Three weeks later, mice were euthanized to analyze the numbers of tumor metastasis nests in the lungs. Figure 4A depicts representative metastatic tumor nests in the lungs from WT, CD27 KO, and CD8Cre-CD27fl groups. We observed a significant increase in metastatic tumor nests in the lungs of CD27 KO mice compared to WT controls or CD8cre-CD27fl mice (Fig. 4C). However, there was no significant difference in metastatic tumor nests between CD8Cre-CD27fl mice and WT controls (Fig. 4B–C). Together, these results indicate that endogenous CD27 signaling in the host also suppresses tumor metastasis via CD8 + T cell-independent mechanisms.

### Global CD27 KO, but not CD8-specific CD27KO, leads to reduced Treg cell frequency and increased B cell frequency in the tumor microenvironment

**Fig. 5:**
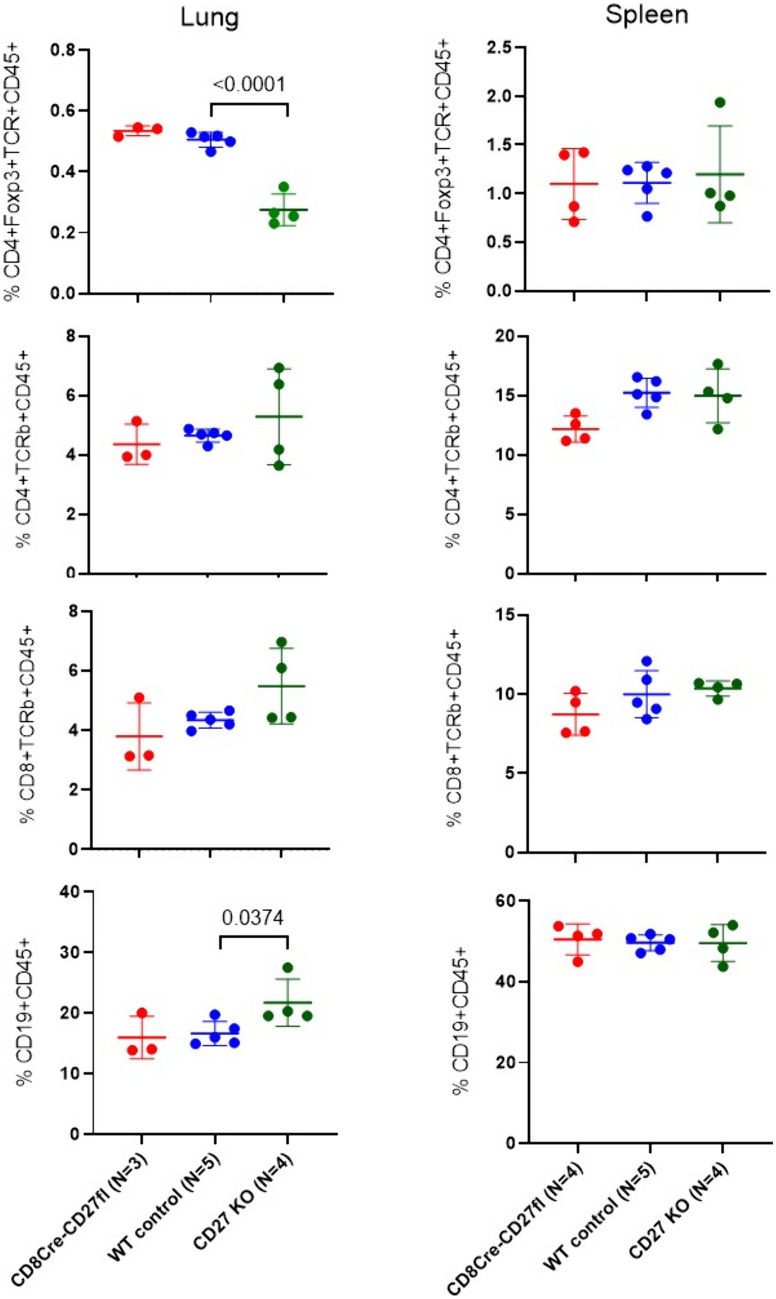
Global CD27 KO, but not CD8-specific CD27KO, leads to reduced Treg cell frequency and increased B cell frequency in the tumor microenvironment. Lung metastasis was developed by intravenous injection of 3 × 10^5^ B16-F10 cells. Ten days after tumor injection, total immune cells were harvested from the lungs after lung tissue disintegration and digestion with collagenase and DNAseI, and then stained with CD45.2, TCRβ, CD19, CD11b, CD4, CD8, and Foxp3, and subjected to flow cytometry analysis. Splenocytes of the tumor bearing mice were also analyzed. Summary data are shown as the frequencies of CD4 + Foxp3 + , CD4 + , CD8 + T cells, and CD19 + B cells within the total live single cells harvested from the lungs and spleen of each mouse (*n* = 3–5 for each genotype). Unpaired T test was performed to determine statistical significance

To assess potential effects on lymphocyte landscape in the tumor microenvironment, we performed flow cytometry to examine the frequencies of major lymphocyte subsets in tumor metastasized lungs ten days after intravenous tumor injection. As shown in Fig. 5, CD8Cre-CD27fl (CD8-specific CD27 KO) did not change the frequencies of CD4 + , CD8 + , CD4 + Foxp3 + T cell subsets or CD19 + B cells in CD45 + immune cells in tumor-infiltrated lungs, while global CD70 KO significantly reduced the frequency of CD4 + Foxp3 + Treg cells, which is consistent with a previous report [6]. Interestingly, global CD70 KO significantly increased the frequency of CD19 + B cells, probably due to reduction of Treg suppression or the increased tumor burden in these mice. In contrast, no significant difference was observed in these lymphocyte subsets in the spleens of these tumor bearing mice (Fig. 5).

### Global CD27 KO mice produced increased cytokines after tumor injection

**Fig. 6:**
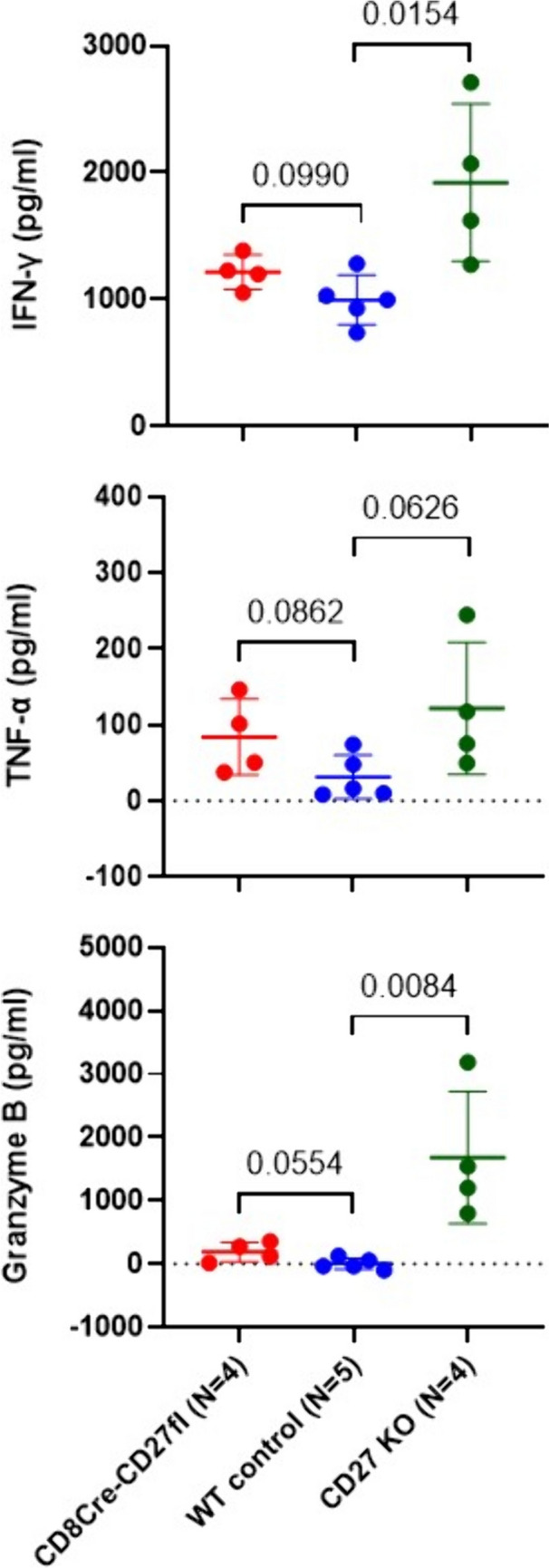
Global CD27 KO mice produced increased cytokines after tumor injection. Lung metastasis was developed by intravenous injection of 3 × 10^5^ B16-F10 cells. Seven days after tumor injection, plasma samples were harvested by retro orbital blood collection. ELISA assays were performed to measure IFN-γ, TNF-α, and Granzyme B in the plasma of each mouse (*n* = 4–5 for each genotype). Unpaired T test was performed to determine statistical significance

To assess potential effects on major effector molecules produced by lymphocytes, we performed ELISA to measure cytokine production in tumor bearing mice seven days after intravenous tumor injection. As shown in new Fig. 6, CD8Cre-CD27fl mice showed a trend of increased production of IFN-γ, TNF-α, and Granzyme B. Interestingly, the global CD27 KO mice showed significantly increased levels of IFN-γ and Granzyme B production, along with an increased trend of TNF-α production. This pattern is associated with increased lung tumor metastasis rates in the global CD27 KO mice, suggesting that increased tumor burden induced higher levels of cytokines in these mice. On the other hand, it is also possible that reduced levels of Treg cells in the tumor microenvironment alleviated immune suppression of other lymphocytes, which produced higher levels of effector molecules via CD27-independent mechanisms. Nevertheless, these data suggest that CD8-specific or global CD27 KO does not reduce the production of these effector molecules.

## Discussion

CD27 has been identified as a co-stimulatory receptor vital for optimal T cell priming and memory differentiation [18, 19]. In many tumors, CD27 remains expressed on infiltrating lymphocytes and can transmit signals to relevant T and NK cells. CD27 signals comprise a major component of the ‘help’ provided to CD8 + T cells from CD4 + T cells via activated antigen presenting cells that express CD70 [5]. Therefore, targeting CD27 may offer significant opportunities to treat various cancers [4, 6]. Modulating CD27-CD70 interaction has been suggested as an attractive strategy to treat solid tumors and hematologic malignancies [20, 21]. An early study using a lymphoma model highlighted that in the activation of cytotoxic CD8 + T cells, CD27 signaling played a central role for antitumor response [22]. In addition, monoclonal antibody-based stimulation of CD27 on T and NK cells increases chemokine and Interferon-gamma release, which elicits myeloid infiltration and macrophage activation, contributing to antitumor efficacy [23]. However, recent studies with diverse tumor and transplant models have described more complicated or even conflicting roles for CD27 in T cell-mediated immune responses [6–11]. CD27 signaling can either improve T cell function or lead to T cell dysfunction, depending on the duration and conditions of receptor ligation [24]. This complex context provided a strong rationale for us to further investigate the role of CD27 expressed on CD8 + T cells, equipped with newly developed cell type-specific conditional KO mice as well as global CD27 KO mice. We chose the commonly used B16-10 melanoma model because of the established methods to study tumor growth and metastasis.

Our results have unveiled that, while endogenous CD27 signaling in the host is important for antitumor immune response, specific deletion of CD27 from CD8 + T cells does not change tumor growth and metastasis in this commonly used melanoma model. At first, our findings seem to contradict with a previous report that CD27-CD70 interaction increased the frequency of Treg cells, reduced tumor-specific T cell responses, and promoted tumor growth [6]. Notably, the previous study mainly inoculated small fragments of tumors (1 mm^3^) including B16-F10, while single cell suspension was injected in our studies for both tumor growth and metastasis. It is likely that the different modes of tumor implantation may lead to the development of different tumor immune microenvironment. Specifically, tumor fragment implantation may have attracted immune profiles in which CD27-expressing Treg cells play a major role in dampening antitumor response. In our model, tumor inoculation in single cell suspension may favor infiltration of CD27-expressing effector lymphocytes that protect against tumor growth and metastasis in a CD27-dependent fashion. Nevertheless, this discrepancy, presumably due to different tumor immune microenvironment, again reveals the intricacy of this pathway in immune response. That is, different timing and tuning of CD27-CD70 signal strength in the context of other T cell receptor and co-receptor signals may collectively tip the balance between immune activation and suppression [25].

Despite the fact that the majority of CD8 + T cells express CD27 protein (Fig. 2), our results show that CD8 + T cells are dispensable in this CD27-dependent antitumor response. It has been shown that tumor-specific T cells are frequently induced naturally in melanoma patients and infiltrate tumors [26]. It is enigmatic why these patients fail to experience tumor regression. Most tumor-infiltrating CD8 + T cells are in the early effector memory stage of differentiation, co-expressing CD27, CD28, CD57, and Granzyme B, with little or no perforin. This population resembles that found in patients with uncontrolled chronic viral infection, suggesting an incompletely differentiated phenotype [26]. Interestingly, our findings also suggest that CD8 + T cells are not activated as effectively as other CD27-expressing lymphocytes, which significantly inhibit tumor growth and metastasis in a CD27-dependent fashion. We postulate that the tumor-inhibiting activity could be due to CD4 + T cells or NK cells. Our data show that more than 90% of CD4 + T cells express CD27 protein (Fig. 2). CD4 + T cells can target tumor cells in various ways, either directly by eliminating tumor cells through cytolytic mechanisms or indirectly by modulating the tumor microenvironment. In a mouse model of therapeutic vaccination, a combination of antibody-based CD27 agonism and PD1 inhibition recapitulated the effects of CD4 + T cell help in promoting CD8 + CTL response [27]. Aside from CD4 + T cells, NK cells could have also contributed to CD27-dependent antitumor activity observed in our model (Figs. 3, 4). Most peripheral blood human NK cells are CD27lo/CD56dim cells, whereas the minor CD27hi NK cell population correspondingly shown a CD56 bright phenotype [28]. It remains to be determined whether human and mouse NK cells, with distinction of CD27lo and CD27hi expression, exhibit CD27-dependent antitumor activity via typical cytotoxicity or cytokine production. Further studies, especially with CD4 + T cell- and NK cell-specific CD27 KO models, are required to determine if these two major CD27-expressing cell types contribute to CD27-dependent antitumor immune response.

In conclusion, this study demonstrates that endogenous CD27 signaling in the host inhibits tumor growth and metastasis via CD8 + T cell-independent mechanisms in the B16-F10 melanoma model, presumably through stimulating antitumor activities of other types of immune cells. Further studies with diverse tumor models are required to determine what cell types contribute to CD27-dependent tumor immunity.

## Data Availability

Data generated during this study are available from the corresponding author upon reasonable request.

## References

[1] Lutfi F et al (2021) Targeting the CD27-CD70 pathway to improve outcomes in both checkpoint immunotherapy and allogeneic hematopoietic cell transplantation. Front Immunol 12:715909 34630390 10.3389/fimmu.2021.715909 PMC8493876

[2] van Lier RA et al (1987) Tissue distribution and biochemical and functional properties of Tp55 (CD27), a novel T cell differentiation antigen. J Immunol 139(5):1589–1596 2442250 10.4049/jimmunol.139.5.1589

[3] Nolte MA et al (2009) Timing and tuning of CD27-CD70 interactions: the impact of signal strength in setting the balance between adaptive responses and immunopathology. Immunol Rev 229(1):216–231 19426224 10.1111/j.1600-065X.2009.00774.x

[4] Bullock TN (2017) Stimulating CD27 to quantitatively and qualitatively shape adaptive immunity to cancer. Curr Opin Immunol 45:82–88 28319731 10.1016/j.coi.2017.02.001 PMC5449212

[5] Buchan SL, Rogel A, Al-Shamkhani A (2018) The immunobiology of CD27 and OX40 and their potential as targets for cancer immunotherapy. Blood J Am Soc Hematol 131(1):39–48 10.1182/blood-2017-07-741025 29118006

[6] Claus C et al (2012) CD27 signaling increases the frequency of regulatory T cells and promotes tumor growth. Can Res 72(14):3664–3676 10.1158/0008-5472.CAN-11-2791 22628427

[7] Muth S et al (2022) CD27 expression on Treg cells limits immune responses against tumors. J Mol Med 100:439–449 34423375 10.1007/s00109-021-02116-9 PMC8843905

[8] Oba T et al (2020) A critical role of CD40 and CD70 signaling in conventional type 1 dendritic cells in expansion and antitumor efficacy of adoptively transferred tumor-specific T cells. J Immunol 205(7):1867–1877 32848036 10.4049/jimmunol.2000347 PMC7511447

[9] Yamauchi T et al (2022) CD40 and CD80/86 signaling in cDC1s mediate effective neoantigen vaccination and generation of antigen-specific CX3CR1(+) CD8(+) T cells. Cancer Immunol Immunother 71(1):137–151 34037810 10.1007/s00262-021-02969-6 PMC8715856

[10] Leigh ND et al (2017) Host-derived CD70 suppresses murine graft-versus-host disease by limiting donor T cell expansion and effector function. J Immunol 199(1):336–347 28550198 10.4049/jimmunol.1502181 PMC5503479

[11] O’Neill RE et al (2017) T cell-derived CD70 delivers an immune checkpoint function in inflammatory T cell responses. J Immunol 199(10):3700–3710 29046346 10.4049/jimmunol.1700380 PMC5687300

[12] Tibbs E et al (2023) Murine regulatory T cells utilize granzyme B to promote tumor metastasis. Cancer Immunol Immunother 72(9):2927–2937 36826509 10.1007/s00262-023-03410-w PMC10690887

[13] Coquet JM et al (2013) Epithelial and dendritic cells in the thymic medulla promote CD4+Foxp3+ regulatory T cell development via the CD27-CD70 pathway. J Exp Med 210(4):715–728 23547099 10.1084/jem.20112061 PMC3620350

[14] Tibbs E, Cao X (2022) Murine myeloid derived suppressor cells possess a range of suppressive mechanisms—Granzyme B is not among them. Cancer Immunol Immunother 71(9):2255–2266 35129637 10.1007/s00262-022-03162-z PMC10693915

[15] Gomez-Cadena A et al (2016) Immune-system-dependent anti-tumor activity of a plant-derived polyphenol rich fraction in a melanoma mouse model. Cell Death Dis 7(6):e2243–e2243 27253407 10.1038/cddis.2016.134 PMC5143373

[16] Jannu AK et al (2021) Lithocholic acid-tryptophan conjugate (UniPR126) based mixed micelle as a nano carrier for specific delivery of niclosamide to prostate cancer via EphA2 receptor. Int J Pharm 605:120819 34166727 10.1016/j.ijpharm.2021.120819

[17] Puppala ER et al (2022) Perillyl alcohol attenuates rheumatoid arthritis via regulating TLR4/NF-κB and Keap1/Nrf2 signaling pathways: a comprehensive study onin-vitro and in-vivo experimental models. Phytomedicine 97:153926 35030388 10.1016/j.phymed.2022.153926

[18] Van De Ven K, Borst J (2015) Targeting the T-cell co-stimulatory CD27/CD70 pathway in cancer immunotherapy: rationale and potential. Immunotherapy 7(6):655–667 26098609 10.2217/imt.15.32

[19] Hendriks J et al (2000) CD27 is required for generation and long-term maintenance of T cell immunity. Nat Immunol 1(5):433–440 11062504 10.1038/80877

[20] Flieswasser T et al (2022) The CD70-CD27 axis in oncology: the new kids on the block. J Exp Clin Cancer Res 41(1):1–15 34991665 10.1186/s13046-021-02215-y PMC8734249

[21] Wajant H (2016) Therapeutic targeting of CD70 and CD27. Expert Opin Ther Targets 20(8):959–973 26914723 10.1517/14728222.2016.1158812

[22] French RR et al (2007) Eradication of lymphoma by CD8 T cells following anti-CD40 monoclonal antibody therapy is critically dependent on CD27 costimulation. Blood J Am Soc Hematol 109(11):4810–4815 10.1182/blood-2006-11-057216 17311995

[23] Turaj AH et al (2017) Antibody tumor targeting is enhanced by CD27 agonists through myeloid recruitment. Cancer Cell 32(6):777–791 29198913 10.1016/j.ccell.2017.11.001 PMC5734932

[24] Libregts S et al (2011) Function of CD27 in helper T cell differentiation. Immunol Lett 136(2):177–186 21277898 10.1016/j.imlet.2011.01.008

[25] Nolte MA et al (2009) Timing and tuning of CD27–CD70 interactions: the impact of signal strength in setting the balance between adaptive responses and immunopathology. Immunol Rev 229(1):216–231 19426224 10.1111/j.1600-065X.2009.00774.x

[26] Wu RC et al (2012) Detection and characterization of a novel subset of CD8+ CD57+ T cells in metastatic melanoma with an incompletely differentiated phenotype. Clin Cancer Res 18(9):2465–2477 22307139 10.1158/1078-0432.CCR-11-2034 PMC3343210

[27] Borst J et al (2018) CD4+ T cell help in cancer immunology and immunotherapy. Nat Rev Immunol 18(10):635–647 30057419 10.1038/s41577-018-0044-0

[28] Silva A et al (2008) Application of CD27 as a marker for distinguishing human NK cell subsets. Int Immunol 20(4):625–630 18326863 10.1093/intimm/dxn022

